# Use of Nonspecific, Glutamic Acid-Free, Media and High Glycerol or High Amylase as Inducing Parameters for Screening *Bacillus* Isolates Having High Yield of Polyglutamic Acid

**DOI:** 10.1155/2014/608739

**Published:** 2014-08-07

**Authors:** Nandita N. Baxi

**Affiliations:** Department of Microbiology and Biotechnology Centre, Faculty of Science, M.S. University of Baroda, Vadodara 390002, India

## Abstract

Out of fifty-five *Bacillus* isolates obtained from ten different regional locations and sources, seven showed the ability to consistently produce specific extracellular polymeric substance (EPS) on rich as well as synthetic but nonspecific media which did not contain glutamic acid. The isolates were identified as either *Bacillus licheniformis* or *Bacillus subtilis*. The EPS from all isolates was resistant to alpha protease, proteinase K, and was thus of high molecular weight. Further it was detected after SDS-PAGE by methylene blue but not by coomassie blue R staining as in case of proteins with high proportion of acidic amino acids. Cell-free EPS, after acid hydrolysis, showed absence of carbohydrates and presence of only glutamic acid. Thus the native the EPS from all seven isolates was confirmed to be gamma polyglutamic acid (PGA) and not exopolysaccharide. The *Bacillus* isolate T which produced maximum polymer on all media tested had higher amylase: protease activity as compared to other strains. If inoculum was developed in rich medium as compared to synthetic medium, the PGA produced increased by twofold in the subsequent synthetic production medium. Similarly, use of inoculum consisting of young and vegetative cells also increased the PGA production by twofold though amount of inoculum did not affect yield of PGA. Though PGA was produced in even in the absence of glutamic acid supplementation in the production medium by all isolates, the yield of PGA increased by fourfold in the presence glutamic acid and the maximum yield was 30 g/l for isolate K. The supplementation of glutamine instead of glutamic acid into the medium caused an increase in the viscosity of the non-Newtonian solution of PGA.

## 1. Introduction

The extrapolymeric substance (EPS) produced by* Bacillus subtilis *and* Bacillus licheniformis *have carbohydrate components as in levan (fructan) or peptide component as in gamma polyglutamic acid (PGA) [1–5]. Although PGA-producing* Bacillus *isolates have been reported many of these produced exopolysaccharide also. Extracellular polymeric substances (EPS) are produced by microorganisms in nature [6, 7] to serve different functions such as biofilm formation, resistance to desiccation, high salinity, and other stress. However,* in vitro, *microbial EPS have been produced using specific/selective media, induced using high carbon substrate, other nutrient ratio, or in presence of specific nutrients and at specific incubation condition of temperature, pH [8]. The present study shows that* amongst EPS-producing Bacillus species isolated from the environment, the PGA-producing isolates are more frequently obtained as compared to isolates that produce polysaccharide substance, *if isolated/screened using rich or synthetic but nonselective media using samples from various locations. In literature the PGA-producing strains are reported to be of two types, either glutamic acid-dependent or independent but ones which are glutamic acid independent PGA producers are adversely affected if glutamic acid or other related components are present in medium [9, 10]. The strains isolated and used in the present study however are not glutamic acid dependent for their ability to produce only PGA type of EPS and are not affected even in presence of glutamic acid or glutamine, in solid media and broth media containing various substrates and are thus suitable for low cost PGA production. The importance of PGA in pharmaceutical products, food industry, and wastewater treatment is well established [9, 11, 12].

## 2. Materials and Methods

### 2.1. Bacteria

*Bacillus* strains were isolated from soil samples collected from various locations in Gujarat, India: hot water spring, oil well, desert, petrol pump, garden, saline creek region, salt pan region, from crude oil, effluent of dye industry, and untreated domestic sewage. Fermented flour samples of soyabean and Bengal gram (threefold increase in batter volume after 18 h after addition of sterile water in sterile container) were also used for isolation of* Bacillus *strains. All samples were suspended in saline and vortexed, and supernatants were heated to 80°C for 15 minutes to kill most of the vegetative, nonsporulating microbial cells and then used to isolate* Bacillus* cultures (colonies) on Luria agar. Morphology of cells of pure cultures of isolates was observed microscopically (1000x) at 6 h–48 h, after inoculating into broth followed by Gram staining, capsule staining (Maneval's method), and endospore staining (Schaeffer and Fulton method) according to standard procedures [13]. The identification of selected mucoid/EPS-forming* Bacillus-*type colonies was determined by BLAST analysis of 16S rDNA partial sequences of isolates with sequences in GENBANK. An accession number was obtained for* Bacillus* T. by submission of sequence to GENBANK.

### 2.2. Media

Several solid media used for growth of EPS producers were Luria Bertanii, soyabean meal, and synthetic media with different substrates: 5–20 g/l of sodium citrate (with 40 g glycerol), glucose, lactose, starch, or skimmed milk. The synthetic basal (Bushnell and Haas) medium used contained (in g/l) MgSO_4_ 0.2, CaCl_2_ 0.02, FeCl_3_ 0.05, K_2_HPO_4_ 1, KH_2_PO_4_ 1, and NH_4_NO_3_ 1. pH was 7.2 ± 0.2. The production medium was basal synthetic medium containing 20 g sodium citrate/l with 40 g glycerol/l as reported in literature [2]. All the media were autoclaved at 10 psi for 20 min.

### 2.3. EPS Analysis

For detection of components of EPS, it was harvested from solid medium in order to rule out contamination of medium constituents in the EPS. Cell-free EPS was hydrolysed using 6 N hydrochloric acid at 110°C either for 2 h in autoclave (10 psi) or for 18 h in oil bath. The hydrolysate was neutralized and the products were analysed by chromatography. Solvent system is composed of n-butanol: acetic acid: water (9 : 6 : 5). Detection reagents used were ninhydrin reagent for amino acids and paraanisidine phthalate for carbohydrates.* After concluding that the EPS was PGA*, native PGA was visualised on SDS-PAGE gel by specific staining with methylene blue because PGA consists of only acidic amino acids [14] and silver staining [15].

### 2.4. PGA Production

For study of accumulation of EPS, biomass of culture priorly grown on rich inoculum media or seed production media was centrifuged at 10,000 ×g for 10 min, washed, and resuspended into fresh production medium (broth). After removal of biomass from broth, EPS was extracted using 3x volumes of acetone and subsequently dried at 60°C. Yield of PGA was calculated as average dry weight (g) of polymer/l of broth. Viscosity of PGA was measured using Brookfield DVII+ viscometer and small sample adapter at different spindle speed.

### 2.5. Enzyme Assay

*Bacillus subtilis *(nonmucoid strain) and mucoid isolates* Bacillus *T and F were inoculated in 1% starch broth or skimmed milk broth and incubated at 37°C. The extracellular amylase activity was assayed using 1% starch substrate and phosphate buffer and the product (reducing sugar) was estimated after 10 minutes using reaction with DNSA reagent and absorbance measured at 540 nm using maltose standard. The extracellular protease activity was assayed using BSA as substrate and by precipitating the residual protein using trichloroacetic acid and the product (amino acids) was estimated using Folin Ciocalteau reagent and absorbance measured at 660 nm using tyrosine as standard.

## 3. Results and Discussion

PGA-producing* Bacillus *isolates have been reported but many of these produced exopolysaccharide also as in the case of natto producing* Bacillus *strains produced EPS consisting of a mixture of PGA and polysaccharide while fermentation of soybeans [16].* Bacillus amyloliquefaciens *was reported to produce more PGA only if mutants with depressed biofilm (polysaccharide) production ability were used [17]. PGA has several established/potential applications in pharmaceutical, food, agriculture industry [9] whereas specific levans of* Bacillus *may have properties similar to exopolysaccharides of other bacterial genera (xanthan, gellan, curdlan, levan, and dextran) [8]. Thus it is important to select* Bacillus* strains that produce either PGA or exopolysaccharide not a mixture of both as it will complicate product recovery and further purification of specific product.

It is known that high carbon: nitrogen ratio of medium is used for exopolysaccharide production and high glutamic acid in medium induces PGA formation but in both cases the producer culture is usually found to produce more than one type of polymer later in other media. Thus in this study only rich and synthetic but nonselective media (media containing neither glutamic acid nor specific carbohydrate for selection) were used for primary screening and isolation of EPS-producing* Bacillus* strains from ten different ecological locations of Gujarat and also from fermented food batter. Since the samples were heated at 80°C for ten minutes, only endospore-bearing cells were isolated as colonies. Out of 56 Bacillus isolates obtained (40 from soil from regions of different petrol pumps, oil wells, hot water springs, desert, gardens, and saline creek; 2 from industrial wastewater; 2 from domestic sewage; 2 from seawater; 10 from fermented flours/beans) only 7 (4 from soils near petrol pumps or oil wells or desert; 1 from sea water, 2 from fermented batter) showed mucoid/highly mucoid colonies on rich media, Luria Bertanii, and subsequently screened using soybean meal medium and on synthetic media containing citrate with glycerol. Morphologically, the colonies were irregular and mucoid and microscopic observation showed presence of Gram positive rods in chains or single, and at a definite stage in growth cycle the cells also showed presence of endospores and capsule. Partial identification using biochemical tests followed by comparison of partial 16S rDNA sequence of six of the EPS-forming isolates with sequences available in GENBANK was done. This leads to the conclusion that six isolates were* Bacillus licheniformis *and one was* Bacillus subtilis*, all from different locations (Table 1). The accession number obtained for* Bacillus licheniformis* T. is JN885459.

**Figure 1:**
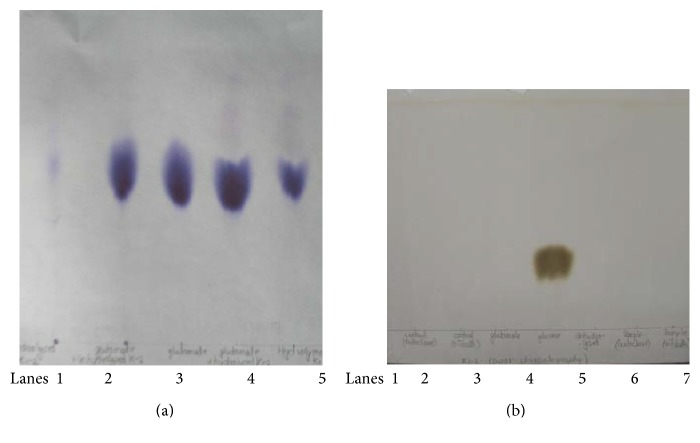
Identification of components of EPS from* Bacillus *K. The polymer (EPS) was obtained from medium without glutamic acid supplementation, medium: 20 g sodium citrate/l, 40 g glycerol/l, in Bushnell and Hass basal medium. EPS was hydrolysed using 6 N HCl at specified conditions and then neutralized with NaOH. Chromatograms were developed using butanol : acetic acid : water (9 : 6 : 5). (a) Detection of components using ninhydrin reagent. Lanes 1 = native polymer, 2 = native polymer + glutamic acid (cochromatography), 3 = standard glutamic acid, 4 = polymer hydrolysed with acid in autoclave at 110°C for 2 h + glutamic acid (cochromatography), 5 = acid-hydrolysed polymer. (b) Detection of components using anisidine phthalate reagent. Lanes 1 = polymer heated without acid at 110°C for 18 h, 2 = polymer heated without acid in autoclave at 110°C for 2 h, 3 = standard glutamic acid, 4 = standard glucose, 5 = native polymer, 6 = polymer hydrolysed with acid at 110°C for 18 h, 7 = polymer hydrolysed with acid in autoclave at 110°C for 2 h.

**Figure 2:**
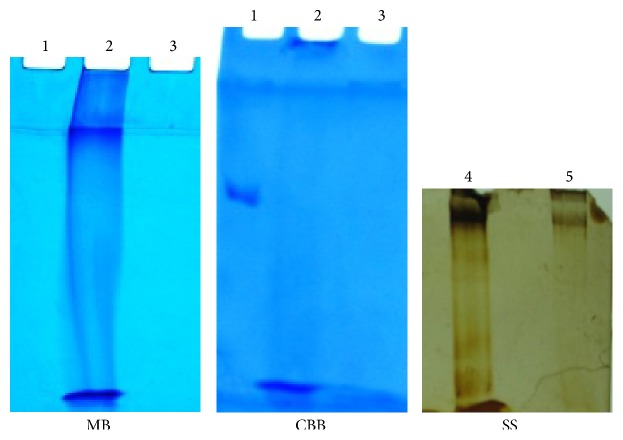
SDS-PAGE of EPS from* Bacillus *K. SDS-PAGE of native and hydrolysed EPS, using 5% stacking and 10% resolving gel and detection by staining with coomassie brilliant blue (CBB) or methylene blue (MB) or silver stain (SS). Stacking gel portion was retained for lanes 1, 2, and 3. Lanes: 1 = bovine serum albumin; 100 microgram, 2,4 = 1000 microgram. 5 = 10 microgram; 3 = acid-hydrolysed polymer.

For confirmation of the nature of EPS of all the seven isolates, EPS from cells grown on solid medium was extracted so as to rule out carryover contamination of medium constituents. Such* cell free of *EPS was hydrolysed using acid and monomers obtained in hydrolysed EPS were identified by chromatography (Figure 1). Surprisingly all showed the presence of only glutamic acid (Rf = 0.5, only sample as well as cochromatography with pure glutamic acid), indicating that the EPS is polyglutamic acid. The absence of carbohydrate (glucose standard was detected as positive control) in the hydrolysate indicated that the EPS is not an exopolysaccharide.* This study thus proves that amongst Bacillus strains isolated from nature, PGA-type of EPS producers is more predominant than exopolysaccharide producers. *The formation of such PGA-type of EPS was not a feature of all the capsulated* Bacillus *isolates, as isolate P was capsulated but nonmucoid and did not yield any extractable polymer. When further visualization of PGA from all 7 isolates was done by SDS-PAGE (Figure 2) only methylene blue (cationic dye) but not coomassie blue could detect the native polymer. PGA consists of only acidic amino acids and thus does not bind to coomassie brilliant R or G [18]. Conversely, standard bovine serum albumin was not easily detected by methylene blue staining. The general method of detection by silver staining is used less frequently by other workers for PGA detection. However in the present study both the methods of staining were found to be equally successful for PGA detection, though detection with methylene blue is more conclusive for PGA. The PGA band obtained using unhydrolysed sample indicated the polymer was of high molecular weight and polydisperse as indicated by smear obtained if high concentration of PGA is used and concentrated staining near high molecular weight location. This is reported in other studies [9] also but our study showed that using a dilute sample, the predominant molecular weight can be more distinctly visualized (Figure 2 lane 5). As expected, the acid-hydrolysed PGA, consisting of only glutamic acid was not detected by SDS-PAGE. The native PGA was found to be resistant to protease action of proteinase K, an alpha protease (standard bovine serum albumin was used as usual substrate for positive control of activity of proteinase K) and this further proved that the EPS was PGA with gamma linkage.

EPS production by three of the* Bacillus licheniformis* isolates K, T, and F was further studied and screened using synthetic media with glucose, starch, and skimmed milk as nutrient source individually or in combination and with a range of pH and sodium chloride, keeping in mind the potential industrial applications [8, 9, 11]. Both of the isolates were found to be suitable for EPS production. In case of media containing only starch as carbon source, though starch was utilized (amylase activity detected), supplementation of minimum amount of glucose (1–2.5 g/l) was required for production of EPS (Table 2). The probable reason for obtaining strains which are able to produce EPS consistently on various media was that initially, for isolation/primary screening, a general, nonspecific synthetic or rich medium was used, instead of specific media containing high carbon: other nutrient ratio or media containing specific nutrients, as recommended for EPS producers [3, 8]. A comparison of relative extracellular amylase : protease activity of mucoid* Bacillus* isolates and nonmucoid* Bacillus subtilis *strain revealed that a higher ratio was found in PGA-producing* Bacillus licheniformis* strain T which produced PGA on all media tested, as compared to strain F or strain* Bacillus subtilis* natto, which produced PGA only in some media. In laboratory conditions it is the medium (nutrient) composition and nutrient utilization which is the inducing factor for polymer production. Other workers have studied the intracellular racemase activity of interconversion of L and D glutamic acid as a parameter to correlate its level with PGA yields. However no earlier studies have reported the use of relative amylase : protease activity (extracellular) for PGA production though this directly indicates nutrient utilization.

For shake flask production, media containing citrate and glycerol as reported in literature [2] for maximum production were used after confirming utilization of citrate as carbon source using pH indicator bromothymol blue (blue colour obtained due to increase in pH due to citrate utilization). Combining the results of PGA produced on solid media and broth,* Bacillus *T. was found to be the most consistent using all media tested. The isolates produced up to 20 g/l PGA in synthetic medium containing sodium citrate and glycerol but without glutamic acid. This is similar to the yield reported in literature.

**Figure 3:**
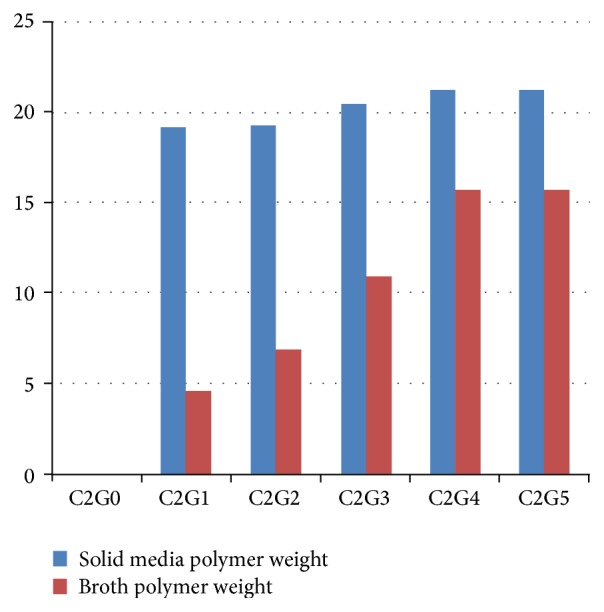
Effect of glycerol on PGA yield by* Bacillus licheniformis *T. glycerol concentration in medium was varied from 0 to 5% (G0–G5) keeping citrate as 2% (C2) in the Bushnell and Hass medium base (pH 7). For solid medium 25 g agar/l was added. Media were inoculated using the same concentration of inoculums. Polymer yield is in g/l.

**Figure 4:**
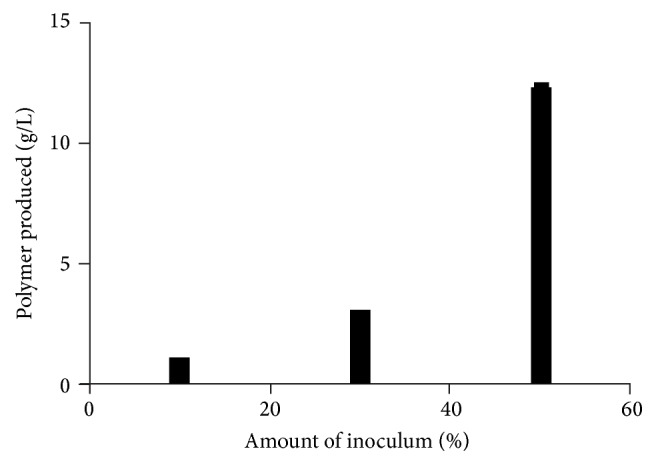
Effect of inoculum on PGA production by* Bacillus licheniformis *T. Inoculum was developed in Luria broth: cells were collected by centrifugation and concentrated from respective volume (10, 30, and 50 mL) by centrifugation, supernatant was discarded and only pellet was used to inoculate production medium (20 g sodium citrate/l, 40 g glycerol/l in Bushnell and Hass medium base pH 7).

As concentration of glycerol in production medium increased the PGA production by* Bacillus licheniformis *was markedly increased in broth but not in solid medium (Figure 3). As expected the increase in inoculums also increased PGA yield (Figure 4). However the important parameter was the physiological status of inoculum of cells which is important for subsequent product formation in most fermentations; PGA production was studied with respect to use of inoculum developed in rich media and synthetic media and use of strictly vegetative cells inoculum (24 h) or a mixture of vegetative and endospore-bearing cells (Table 3(a)). A twofold increase was obtained using inoculum cells grown in rich media as compared to cells grown in synthetic (production) media though use of the same medium for inoculum should minimise adaptation. Similarly a twofold increase in PGA production was obtained using inoculum consisting of young (24 h) vegetative cells as compared to mixture of vegetative cells and sporulated cells (72 h). However the amount of inoculum used (4–20% v/v) did not change the yield of PGA.* Such kind of studies has not been reported for PGA production and these results will lead to PGA production using low cost nutrients but high biomass*.

The isolates used in present study produced only PGA type of EPS. The production of PGA was fourfold higher in presence of glutamic acid (up to 30–40 g/l) as compared to when it was not supplemented in the medium (Table 3(b)). The yield of PGA by the isolates used in this study, in medium containing glutamic acid, was comparable to that reported in literature (wild type/genetically engineered* Bacillus *strains) as 5–100 g/l [9, 17].* But these earlier reports do not clearly mention whether the production of EPS is of only PGA or also exopolysaccharide* by the* Bacillus *strain. Though glutamic acid independent production of PGA from* Bacillus *strain has also been earlier reported to be 22 g/l [10], a very high level of carbon (75 g fructose or glucose/l and 18 g ammonium chloride/l) was required in that case and again there is no clear indication in that report of the production of only PGA or also exopolysaccharide as a mixture. Moreover in this case in the presence of glutamic acid the PGA production was reported to be* decreased* threefold, and such a case may be often prevalent in raw material containing mixture of substrates. The isolates reported in this present study did not need higher amount of carbon source and PGA production is not decreased even if mixture of substrates is present in medium. Thus these isolates were superior from the point of view of PGA product production.

PGA is known to have several applications due to its water retention capacity and also due to its viscosity [9]. The viscosity of broth containing PGA is usually reported to increase and shows non-Newtonian characteristics [19]. Whenever PGA is not the primary product desired, PGA mutants have to be used [20]. In the present study the viscosity broth culture producing PGA increased as PGA accumulated. The broth cultures of* Bacillus *K, R, D, T, and F showed increase in viscosity after 24 h of incubation, visibly indicating accumulation of EPS, but the same was not true for other PGA-producing isolates. Broth containing cells as well as PGA was used to extract PGA using solvent. Subsequently the precipitated PGA* itself* was solubilised in water and used for measurement of viscosity rather than that of the whole broth which would contain mixture of cells along with PGA (Table 4). PGA obtained from isolate T was subjected to varying shear stress and as expected, with an increase in shear rate the viscosity of PGA decreased (thinning effect of non-Newtonian polymer solutions). An interesting observation was that there was a difference in viscosity of PGA solution (fixed concentration) depending on use of either glutamic acid or glutamine in medium used for PGA production by the same* Bacillus *strain.

## 4. Conclusion

The use of rich but nonselective media for isolation of* Bacillus* strains capable of EPS-production leads to isolation of PGA producers capable of producing only high molecular weight, polydisperse, viscous PGA as constituent. The amount and viscosity of PGA produced depend on the relative amylase: protease activity and use of specific medium constituents and PGA was even produced in presence of mixed substrates. These isolates are glutamic acid independent PGA producers which do not produce exopolysaccharide and thus are superior for PGA production to the other wild type or genetically engineered* Bacillus* strains reported earlier in literature.

## Figures and Tables

**Table 1:** Primary screening of *Bacillus *strains producing extracellular polymeric substance (EPS).

Culture	Source^a^	Phenotype of colonies on rich media and synthetic medium^b^	Identification^c^ (16S rDNA and biochemical tests)
*Bacillus* K.	Soil near oil well	Highly mucoid	*Bacillus licheniformis *
*Bacillus *R.	Seawater	Highly mucoid	*Bacillus licheniformis *
*Bacillus *T.	Soil near petrol pump	Highly mucoid	*Bacillus licheniformis *
*Bacillus *D.	Desert soil	Highly mucoid	*Bacillus licheniformis *
*Bacillus *O.	Fermented soybean flour	Mucoid	*Bacillus licheniformis *
*Bacillus *F.	Fermented gram flour	Mucoid	*Bacillus licheniformis *
*Bacillus *B.	Fungus infested field soil	Variable (mucoid)	*Bacillus subtilis *

^a^Isolation of *Bacillus*: samples were suspended in saline and vortexed; supernatants were heated to 80°C for 15 minutes to kill all vegetative, non-endospore bearing cells. Supernatants were used for direct isolation on solid media.

^b^Solid media (without glutamic acid supplementation) for isolation: Luria agar (pH 7–11, NaCl 0–100 g/l) and synthetic medium containing 20 g citrate/l and 40 g glycerol/l (pH 7).

^c^Bacterial isolates were inoculated into Luria broth and incubated for 18 to 48 h. Gram staining, capsule staining (Maneval's method), and endospore (Schaeffer and Fulton method) staining done at various intervals. All isolates showed presence of Gram positive rods in chains or single cells with capsule and endospores at different times and were further identified.

**Table 2:** Extracellular amylase: protease activity and PGA production by *Bacillus* strains.

Parameter/property	*Bacillus licheniformis *T.		*Bacillus licheniformis *F.		*Bacillus subtilis *	
	24 h	48 h	24 h	48 h	24 h	48 h
Amylase^a^ units/mL broth	5000	6744	4200	5995	3900	5578
Protease^b^ units/mL broth	42	50	42	56	42	72
Amylase: protease: 24 h, 48 h	120	134	100	107	93	76
PGA (g/L)^c^ in broth media with sodium citrate + glycerol (10, 40 g/L)		11 (±3.4)		22 (±1.2)		6 (±1.3)
PGA^c^ on solid media containing glucose (10–20 g/L)	+	+	(+)	(+)	−	−
PGA production^c^ on media containing starch (5–20 g/L) and amylase activity^d^ detected	−, A	−, A	−, A	−, A	+, A	+, A
PGA production^c^ on synthetic media containing starch (10 g/L), glucose (2.5 g/L); Amylase^d^ detected	+, A	+, A	−, A	−, A	+, A	+, A
PGA production^c^ on synthetic media containing starch (10 g/L), glucose (2.5 g/L), NaCl (10–100 g/L); Amylase^d^	+, A	+, A	−, A	−, A	+, A	+, A
PGA production^c^ on synthetic media containing skimmed milk 20% v/v, pH 4–8; Protease detected^e^	+, P	+, P	+, P	+, P	+, P	+, P

^a^Amylase/^b^Protease assay**:** *Bacillus  subtilis* (nonmucoid strain) and *Bacillus *T. and F. were inoculated in 1% starch broth or 1% skimmed milk broth and incubated at 37°C and amylase/protease activity was assayed.

^c^Production medium (pH = 7): all substrates were added to Bushnell and Hass synthetic medium base (composition g/L: MgSO_4_ 0.2, CaCl_2_ 0.02, FeCl_3_ 0.05, K_2_HPO_4_ 1, KH_2_PO_4 _1, NH_4_NO_3_ 1, and agar 25.

+/(+) = PGA present/variable. Values in parentheses are the standard deviation. − = PGA absent.

^d^A = amylase activity using starch agar (white/brown zone of hydrolysed starch visible after addition of Lugol's iodine solution).

^e^P = protease activity (clear zone against turbid background of skimmed milk agar).

**(a):** 

Inoculum medium (broth)	Luria Bertanii		Synthetic	
Inoculum age (h)	24–36	72	24–36	72
Vegetative cells present/2 cm^2^ square smear	800	112	360	38
Sporulated cells present/2 cm^2^ square smear	0	920	0	460
Polymer^c^ (g/l) in broth (Inoculum used: 4% v/v)	7.1	4.5	4.5	2.1
Polymer^c^ (g/l) in broth (Inoculum used: 20% v/v)	6.5		5.4	

**(b):** 

Synthetic medium^b^	With glutamic acid (20 g/l)		Without glutamic acid	
PGA producing culture	*Bacillus* K.	*Bacillus *T.	*Bacillus *K.	*Bacillus *T.
PGA on solid medium	+	+	+	+
PGA in broth (g/l)^c^	30 (±4.5)	11 (±3.4)	7 (±1.1)	2.5 (±1.1)

^a^Polymer was either observed on solid medium as mucoid colonies (+) or extracted from broth cultures incubated on rotary shaker (180 rpm) using 3x volumes of acetone. Values are of dry weight of precipitated polymer, SD in parentheses.

^b^Production medium: 20 g sodium citrate/l, 40 g glycerol/l in Bushnell and Hass medium base (pH 7). For solid medium 25 g agar/l was added.

^c^For broth culture, 50 mL medium in 250 mL Erlenmeyer flasks was inoculated (6% inoculum v/v after centrifugation and discarding supernatant) with culture (OD = 1) and incubated for 24 to 96 h at 30°C. Polymer was extracted using 3x volumes of acetone, dried at 60°C.

**Table 4:** Viscosity^a^ of polyglutamic acid^b^ from *Bacillus* K.

Rpm of spindle	Viscosity of polymer (1.4%)		
	Medium^b^ + 20 g sodium glutamate/L		Medium^b^ + 20 g glutamine/L
	+NH_4_NO_3_	−NH_4_NO_3_	+NH_4_NO_3_
5	36	36	60
10	24	30	48
50	2.4	3.4	39.6
100	0	0	34.8

^a^Viscosity (centipoise = 0.01 poise gm^−1^ cm^−1^ s) of the broth was measured at 30°C using Brookfield DV II + viscometer using a small sample adapter at a varying spindle speeds. Xanthan gum (0.25%) was used as standard for viscosity (20–60 cp) at various rpm (5–100). ^b^Polymer was extracted from broth or solid medium (medium: sodium citrate 20 g/L and glycerol 40 g/L, in Bushnell and Hass medium. Glutamic acid supplemented only where mentioned).

Extraction was done using 3 volumes of acetone and dried at 60°C. Redissolved polymer (fixed concentration) was used for viscosity measurement.
